# COmmunity-based Rehabilitation after Knee Arthroplasty (CORKA): statistical analysis plan for a randomised controlled trial

**DOI:** 10.1186/s13063-018-3031-7

**Published:** 2018-11-19

**Authors:** Karan Vadher, Ruth Knight, Karen L. Barker, Susan J. Dutton

**Affiliations:** 1 Oxford Clinical Trials Research Unit, Centre for Statistics in Medicine, Nuffield Department of Orthopaedics, Rheumatology and Musculoskeletal Sciences, University of Oxford, Oxford, OX3 7LD UK; 2 National Institute for Health Research Biomedical Research Unit, Nuffield Department of Orthopaedics, Rheumatology and Musculoskeletal Sciences, University of Oxford, Oxford, OX3 7LD UK; 3 Physiotherapy Research Unit, Nuffield Orthopaedic Centre, Oxford University Hospitals NHS Foundation Trust, Oxford, OX3 7HE UK

**Keywords:** Statistical analysis plan, Randomised controlled trial, Knee arthroplasty, Physiotherapy, Occupational therapy, Rehabilitation, Community, Elderly, Frail

## Abstract

**Background:**

About 15% of patients fail to achieve a satisfactory clinical outcome following knee replacement, which may indicate the existing model of rehabilitation after surgery is possibly not the most efficacious. The COmmunity-based Rehabilitation after Knee Arthroplasty (CORKA) trial evaluates the effects of a new multi-component community-based rehabilitation programme following knee replacement compared with usual care.

**Methods/design:**

The CORKA trial is a multi-centre, single-blind, two-arm randomised controlled trial. The primary outcome is the Late Life Function and Disability Instrument (LLFDI) overall function score measured at 12 months post-randomisation which will be analysed using a linear mixed effects model. Secondary outcomes are measured at 6 and 12 months post-randomisation and include the LLFDI frequency and limitation total dimension scores, the Oxford Knee Score, the Knee injury and Osteoarthritis Outcome Score quality of life subscale, the Physical Activity Scale for the Elderly, the EuroQol EQ-5D-5L, and several measurements of physical function*.* Full details of the planned analysis approaches for the primary and secondary outcomes are described here, as are the descriptive statistics which will be reported. This is an update to the CORKA protocol which has already been published in this journal.

**Discussion:**

This paper provides details of the planned statistical analyses for this trial and will reduce the risks of outcome reporting bias and data-driven results.

**Trial registration:**

ISRCTN registry, 13517704. Registered on 12 February 2015.

**Funding/sponsor:**

The trial is funded by the National Institute for Health Research Health Technology Assessment programme under its commissioned research programme (HTA 12/196/08). The trial sponsor is the University of Oxford.

## Background

Osteoarthritis is the most common cause of disability in older people [1], with painful knee osteoarthritis affecting 10% of people older than 55 in the UK [2]. The number of knee replacements taking place in the UK has risen significantly in recent years; in 2016, 98,147 primary knee replacements were recorded [3]. Outcomes following knee replacement are known to be multi-faceted; around 15% of patients do not report a good outcome from their knee replacement and have continuing pain and mobility problems [4]. Factors such as the amount of pain and limitation of balance and muscle strength may contribute to poorer outcomes [5].

Best practice guidance from the UK and North America recommends that a multi-disciplinary approach to rehabilitation improves outcomes [6–8]; it is believed that such an approach may contribute to optimising post-operative return to functional activity. In addition, it is clear from existing studies that current rehabilitation strategies do not meet the needs of all patients, particularly those who are socially isolated; do not have easy access to transport; or who are frail. A review examining multi-disciplinary rehabilitation programmes following hip and knee joint arthroplasty indicated that home-based care may be beneficial; however, it concluded that further high-quality research is needed [8].

The COmmunity-based Rehabilitation after Knee Arthroplasty (CORKA) trial is a multi-centre, single-blind, two-arm, randomised controlled trial designed to compare the clinical outcomes of a new home-based multi-disciplinary rehabilitation protocol with usual care in patients assessed pre-operatively as being at risk of a poor outcome. The protocol paper for the CORKA trial has been published previously [9]; the aim of this paper is to report in detail the analysis plan as agreed on by the trial steering committee in October 2017.

## Methods/design

### Trial design

CORKA is a multi-centre, single-blind, two-arm, individually randomised controlled, superiority trial with blinded outcome assessment at 6 and 12 months post-randomisation. Randomisation uses a 1:1 ratio, is stratified by study centre, and is performed via a secure, web-based randomisation system. The nature of the intervention means participants and those delivering the intervention are not blinded to treatment allocation; however, clinical outcome assessors remain blinded to treatment allocation where possible. Eligible participants identified pre-operatively as being at risk of a poor outcome using the study screening tool [10] are randomised to receive either a new multi-component community-based rehabilitation programme or usual care. Outcomes are assessed at baseline and at 6 and 12 months post-randomisation. Full details of the trial design, study population, and study procedures have been published previously [9].

The trial is registered with the International Standard Randomised Controlled Trials database, ISRCTN reference number 13517704.

### Objectives

The primary objective of this study is to determine if a multi-component rehabilitation programme improves the outcome at 12 months after surgery of patients who undergo knee replacement. The null hypothesis is that there is no difference in patients’ functional abilities between the two treatment groups. Secondary objectives include assessing the impact of the rehabilitation programme on physical function and quality of life at 6 and 12 months after randomisation, and a cost effectiveness analysis of the new therapy versus usual care rehabilitation up to 12 months after randomisation.

### Outcomes

#### Primary outcome

The primary outcome is the Late Life Function and Disability Instrument (LLFDI) overall function score [11] measured at 12 months post-randomisation. The LLFDI overall function score consists of 32 items, and scores range from 0 to 100 with higher scores indicating better functionality. The LLFDI overall function score is also measured at 6 months post-randomisation.

#### Secondary outcomes

The secondary outcome measures, recorded at 6 and 12 months post-randomisation, are as follows:

- LLFDI frequency and limitation total dimension scores: 16-item patient reported outcome measures (PROMs), each with a total score ranging from 0 to 100, with higher scores indicating higher levels of frequency of participation in life tasks and capability of participation in life tasks respectively.
- The Oxford Knee Score (OKS) [12]: a 12-item PROM with total scores ranging from 0 (most severe symptoms) to 48 (least severe symptoms).
- The Knee injury and Osteoarthritis Outcome Score (KOOS) quality of life subscale [13]: four self-reported questions with total scores ranging from 0 (extreme problems) to 100 (no problems)
- The Physical Activity Scale for the Elderly (PASE) questionnaire [14]: a 12-item, self-reported measure. The time spent participating in each activity is multiplied by a weight corresponding to that activity, and these weighted values are summed to calculate total PASE scores, with higher scores indicating greater levels of physical activity.
- Health economics using the EuroQol EQ-5D-5L [15]: a self-reported outcome measure consisting of 5 dimensions each with 5 possible responses which are converted to multi-attribute utility scores, where 1 represents perfect health, 0 represents death, and scores less than 0, representing a quality of life worse than death, are possible.
- Measurement of physical function using the 30-s chair stand test, the figure of 8 walking test, and the single-leg stance test.

For the subgroup of patients who reach 2 years post-randomisation prior to the end of the trial, questionnaire data will be collected at this time point. This will include the LLFDI overall function score, and frequency and limitation total dimension scores, the OKS, the KOOS quality of life subscale, the PASE, and the EuroQol EQ-5D-5 L.

#### Safety outcomes

The number of adverse events and serious adverse events occurring whilst a participant is continuing in the study up to 12 months post-randomisation will be reported along with the relatedness of these events to the treatment. Serious adverse events are defined as those that are fatal, life threatening, or disabling, or that require hospitalisation or prolongation of hospitalisation.

### Sample size

The primary outcome in the CORKA trial is the LLFDI overall function score at 12 months. No existing information was available about the minimum clinically important difference for this scale; however, it was believed to be the most clinically relevant outcome in this population. Therefore, the sample size calculation was based on a moderately small standardised effect size of 0.275. This standardised effect size is, for example, equivalent to detecting a 3-point difference between treatment arms on the LLFDI overall function score assuming a standard deviation of 10.91 and no clustering effect across centres [16]. Six hundred and twenty participants (310 per arm) are required to detect a standardised effect size of 0.275 with 90% power, 5% (two-sided) significance, and allowing for 10% loss to follow-up based on previous experience of trials in a similar population.

An internal pilot study was conducted at one site (Oxford) to review for recruitment feasibility and to confirm the intervention package. Fifteen patients were randomised during this pilot study, and these patients will be used in the final analysis, since the intervention package was not changed between the pilot and the main trial.

### Statistical analysis

#### General analysis principles

Two analysis populations will be considered: (1) the intention-to-treat (ITT) population; and, (2) the per-protocol (PP) population. The ITT population will include all randomised participants analysed according to the intervention to which they were randomised. The ITT population will be the primary analysis set, and all analyses will be conducted for this population if not otherwise stated. Analyses on this population provide an estimate of the effect of treatment offer.

The PP population will be analysed according to the intervention they actually received. Participants with major protocol deviations or violations, that is, those who did not receive treatment or did not provide any follow-up data, will be excluded from the PP population. The definition of the PP population will be finalised by the trial statistician during a blinded analysis of the data (i.e. without details of treatment allocation) prior to the primary analysis time point; any unanticipated protocol deviations which occur will be identified at this point. Analysis of the PP population will be conducted as sensitivity analyses for important outcomes and will address the effect of the intervention in those who receive it and provide follow-up data.

The significance level used throughout will be 0.05, and 95% confidence intervals will be reported. The primary conclusion of the trial will be based on the results from the primary analysis of the primary outcome, and all secondary analyses will be considered as being supportive of the primary results.

All analyses will be carried out using appropriate, validated statistical software such as Stata [17] or R [18]. The relevant package and version number used for the analysis will be recorded and reported.

#### Descriptive analyses

**Fig. 1:**
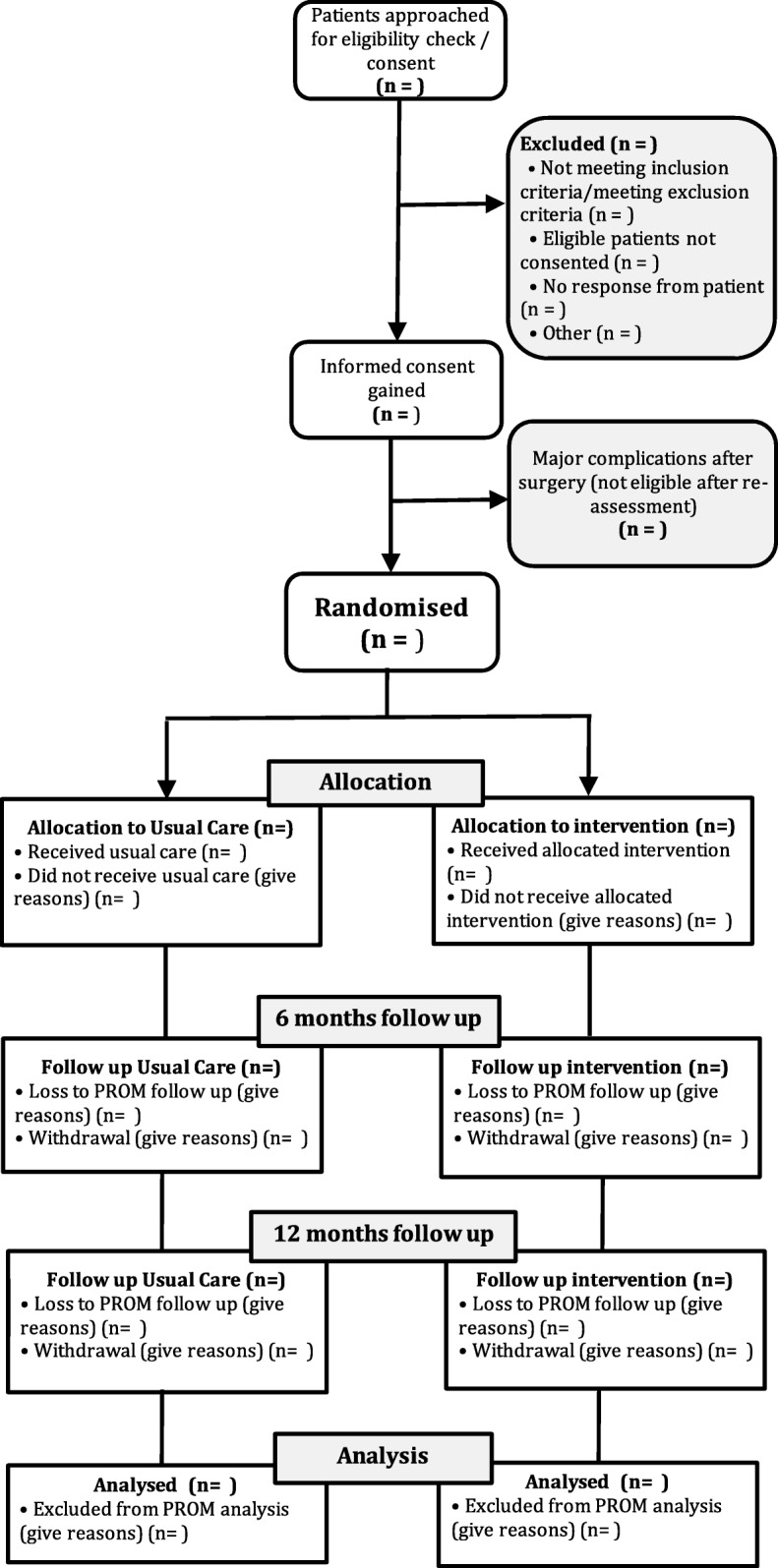
CONSORT flow chart of trial participants

The flow of participants through the trial, including the number of individuals assessed, recruited, randomly assigned to either usual care or multi-component community-based rehabilitation, and analysed for the primary outcome will be summarised using a Consolidated Standards of Reporting Trials patient reported outcome (CONSORT-PRO) extension flow chart [19] (see Fig. 1).

The baseline comparability of the two randomised groups in terms of: (1) risk factors for poor outcome after knee replacement according to the CORKA screening tool [10]: (2) baseline characteristics (Table 1); and, (3) primary and secondary outcomes at baseline will be presented. Numbers with percentages will be used to describe binary and categorical variables, and either means and standard deviations or medians and interquartile ranges, if a variable is not normally distributed, will be used for continuous variables. There will be no tests of statistical significance or confidence intervals for differences between randomised groups.

**Table 1:** Baseline characteristics

Baseline characteristic	Type	Levels or scale
Gender	Categorical	Male; Female
Age	Categorical	55–64 years; 65–74 years; 75 years or more
Age	Continuous	Years
Fallen in the last year	Categorical	Yes; No
Number of falls in last year	Continuous	NA
Previous lower limb surgery	Categorical	Yes; No
Charnley ABC classification	Categorical	Single knee arthroplasty; Both knees affected; Multiple joint disease (or other disability)
Self-reported current mobility (stairs)	Categorical	Normal; One step at a time; Down with rail; Up and down with rail; Unable down; Unable
Self-reported current mobility (support)	Categorical	None; Stick outdoors; Stick always; 2 sticks; 2 crutches; Walking frame
Body mass index	Continuous	kg/m^2^

#### Loss to follow-up, withdrawals, and missing data

Losses to follow-up and withdrawals before the 6 months follow-up and the 12 months follow-up will be reported by intervention arm, together with reasons for the losses. To ensure that there are no differential losses between the two groups, this will be tested using absolute risk differences and a chi-squared test. The patterns of availability of data will be presented by treatment arm and overall for all outcomes from baseline to the end of follow-up. Where appropriate, differentiation will be made between partially completed and fully missing outcome data. Reasons for missing data will also be summarised.

#### Compliance

Attendance will be recorded for the intervention, and compliance with the test intervention will be defined as fulfilling at least four treatment sessions. This is the clinical consensus definition based on variation in current practice and expected effective dose. In addition, this represents participants who have received at least half of the specified intervention.

For primary and key secondary outcomes, a complier average causal effect (CACE) analysis will be estimated using an instrumental variable approach to assess the impact of compliance on the outcomes [20, 21]. This will be a sensitivity analysis for these outcomes, and it will provide an unbiased estimate of treatment effect amongst those who would receive the intervention as planned.

#### Analysis of primary outcome

The LLFDI at 12 months will be summarised by treatment arm using means and standard deviations and analysed using a linear mixed effects model with repeated measures incorporating outcome measurements at 6 and 12 months and adjusting for baseline score and study site as covariates. An interaction between outcome measurement time point and randomised group will be fitted to allow estimation of the treatment effect at each time point, reported as the adjusted mean difference in LLFDI between groups at 12 months along with the standard deviation of this difference; the treatment effect at 12 months will be considered the primary endpoint. The distribution of the change from baseline will be assessed for evidence of departure from normality using residual plots. If necessary, data will either be transformed to achieve normality or analysed using a non-parametric equivalent.

Steps will be taken to minimise missing data, including repeated efforts by the trial team to contact those who do not return for their 12 month appointment; however, there will inevitably be some missing data either in the form of total non-response post-randomisation or item non-response. Missing data will be reported for both types of non-response, and reasons for the non-response will be provided. The missing data pattern will be explored. In order to be consistent with the ITT principle, missing data will be imputed using multiple imputation (MI) techniques [22]. The imputation model used will include all variables used in the analysis model (baseline score and study site) as well as any other important factors believed to predict the outcome or missingness. This judgement will be based on visual assessment and clinical opinion and will improve the validity of the imputation model under the missing at random (MAR) assumption. Imputation will be performed separately for each treatment group, and the number of replicates will be equal to the proportion of missing data [23]. Deviations from the MAR assumptions will be explored by repeating the MI under different assumptions as part of the sensitivity analyses. In the unlikely event of a large number of missing data points for a variable in the primary outcome analysis, an alternative method will be considered during the blinded analysis, as MI is not suitable in this case.

A sensitivity analysis will be carried out on a PP basis to examine the robustness of conclusions to different assumptions about departures from randomised policies.

#### Analysis of secondary outcomes

The LLFDI frequency and limitation total domain scores will be analysed using the methods described for the primary outcome.

If the assumption of normality holds, other continuous outcomes will be summarised using means and standard deviations and analysed using *t* tests, analysis of covariance (ANCOVA), or multi-variate linear regression as appropriate. The analysis will be adjusted for baseline questionnaire score, stratification factor (study site), and other important prognostic factors if either ANCOVA or multi-variate linear regression is used. If the model assumptions are violated, the data will be summarised using medians and interquartile ranges, and either transformed to achieve normality prior to testing or, if this is not possible, tested using the Mann-Whitney *U* test.

Binary and categorical outcomes will be reported as proportions with 95% confidence intervals and will be assessed using chi-squared tests and logistic regression to adjust for potential confounders and baseline covariates. Ordinal logistic regression will be considered for ordered categorical outcomes.

Missing items will be dealt with using the pre-specified techniques each questionnaire recommends. If, for any questionnaire, no technique is pre-specified, mean imputation will be used if more than half the items are completed and the value regarded as missing otherwise. For key secondary outcomes (LLFDI frequency and limitation total domain scores, OKS, and KOOS) sensitivity analyses will be conducted on a PP basis to examine robustness of conclusions. Furthermore, missing data on the key secondary outcomes managed by complete case analysis will be investigated and MI conducted to see how sensitive the estimates are, if appropriate.

#### Safety analyses

The number of adverse events and serious adverse events occurring whilst a participant is continuing in the study will be reported along with the relatedness of these events. A comparison between the two treatment groups of the total number of participants experiencing serious adverse events will be conducted by examination of 95% confidence intervals for differences in incidence.

#### Additional analyses

Factors associated with an improved treatment response will be assessed to see whether a particular patient population is more likely to have a positive response. The distribution of risk factors for poor outcome after knee replacement according to the CORKA screening tool will also be investigated.

For the subgroup of patients who provide 2 year follow-up data, methods previously described to analyse the data from each questionnaire will be repeated incorporating this measurement.

## Discussion

The CORKA trial will provide data regarding the effects of a multi-component community-based rehabilitation programme on the outcomes of patients 12 months after knee replacement surgery, compared to those receiving usual care. This paper provides details of the planned statistical analyses for this trial and will help reduce the risks of outcome reporting bias and data-driven results [24]. This paper has been prepared in accordance with the published guidelines for the content of statistical analysis plans [25].

## Trial status

Recruitment for the trial closed on 26 January 2018. In total 621 patients from 14 study sites were recruited. Follow-up is currently ongoing and expected to finish in January 2019. The final analysis will be conducted thereafter.

## Abbreviations

CACE: Complier average causal effect
ITT: Intention-to-treat
KOOS: Knee injury and Osteoarthritis Outcome Score
LLFDI: Late Life Function and Disability Instrument
MAR: Missing at random
MI: Multiple imputation
OKS: Oxford Knee Score
PASE: Physical Activity Scale for the Elderly
PP: Per-protocol
PROM: Patient reported outcome measure
